# *Sirt1* regulates testosterone biosynthesis in Leydig cells via modulating autophagy

**DOI:** 10.1007/s13238-020-00771-1

**Published:** 2020-10-13

**Authors:** Muhammad Babar Khawar, Chao Liu, Fengyi Gao, Hui Gao, Wenwen Liu, Tingting Han, Lina Wang, Guoping Li, Hui Jiang, Wei Li

**Affiliations:** 1 State Key Laboratory of Stem Cell and Reproductive Biology, Institute of Zoology, Chinese Academy of Sciences, Beijing, 100101 China; 2 School of Biotechnology and Food, Shangqiu Normal University, Shangqiu, 476000 China; 3 University of Chinese Academy of Sciences, Beijing, 100049 China; 4 The MOH key Laboratory of Geriatrics, Beijing Hospital, National Center of Gerontology, Beijing, 100730 China; 5 Department of Urology, Peking University Third Hospital, Beijing, 100191 China; 6 Department of Andrology, Peking University Third Hospital, Beijing, 100191 China; 7 Department of Reproductive Medicine Center, Peking University Third Hospital, Beijing, 100191 China; 8 Department of Human Sperm Bank, Peking University Third Hospital, Beijing, 100191 China

**Dear Editor,**

Steroid hormones are crucial signal molecules that regulate a large number of physiological and developmental processes. Testosterone is the key steroid hormone required for the development of male characteristics and also supports the physiology of the male reproductive system (Sinclair et al., 2015). Testosterone is primarily produced by the Leydig cells residing in the testicular interstitium. The cholesterol acts as a substrate for the biosynthesis of testosterone. Since steroidogenic cells are capable of storing only very little hormone, rapid synthesis of hormone requires the mobilization of the precursor cholesterol, chiefly stored as intracellular lipid droplets (LDs) (Danielsen et al., 2016). Leydig cells are the major sites to produce testosterone, there are extremely active autophagy in them, and a decline in steroidogenesis has also been associated with the decline of autophagic flow. Moreover, the disruption of autophagy leads to decreased intracellular LDs, and therefore affects testosterone synthesis in the Leydig cells (Danielsen et al., 2016; Gao et al., 2018).

Sirtuin 1 (SIRT1) is one of the members of NAD-dependent protein deacetylase which regulates multiple cellular functions such as metabolism, apoptosis, and autophagy (Cohen et al., 2004; Chang et al., 2015). SIRT1 is necessary for fertility in mice, it participates in the differentiation of spermatogenic stem cells, acrosome biogenesis, and histone-to-protamine transition during spermatogenesis (Liu et al., 2017). However, the functional role and underlying mechanism of SIRT1 in testosterone biosynthesis are yet unknown.

To explore the role of *Sirt1* in testosterone biosynthesis, *Sirt1*^*F*/*F*^ mice were crossed with *SF1-Cre* mice to generate steroidogenic cell-specific *Sirt1*-knockout mice, and the knockout efficiency of *SF1-Sirt1*^−/−^ mice was further confirmed by Western blot (Fig. 2E). We found no difference in testis morphology (Fig. S1A) and the sperm count in cauda epididymis (Fig. S1B) of *Sirt1*^*F*/*F*^ and *SF1-Sirt1*^−/−^ mice. However, the mating efficiency and pregnancy rate of *SF1-Sirt1*^−/−^ mice were significantly decreased compared to *Sirt1*^*F*/*F*^ mice (Fig. S2). To investigate reduced mating efficiency and docile behavior, we carried out sexual behavior analysis. *SF1-Sirt1*^−/−^ mice showed a significantly longer latency (Fig. 1A) and mounted targeted females less frequently and with a shorter mounting duration (Fig. 1B and 1C) compared to the *Sirt1*^*F*/*F*^ mice. However, sniffing frequency and duration was not significantly affected in *SF1-Sirt1*^−/−^ mice compared to control mice (Fig. 1D and 1E). In vertebrates, male sexual behavior is regulated by testosterone, thus we measured the serum testosterone concentration and found reduced testosterone levels in the sera of *SF1-Sirt1*^−/−^ mice (Fig. 1F). Testosterone levels were also significantly reduced in the Leydig cells of *SF1-Sirt1*^−/−^ mice (Fig. 1G). Together, these findings suggest that *Sirt1*-disruption results in a sharp decrease in testosterone and influence the sexual behavior of male mice, which is very similar to the symptoms of late-onset hypogonadism (LOH) (Swee and GAN, 2019).

**Figure 1:**
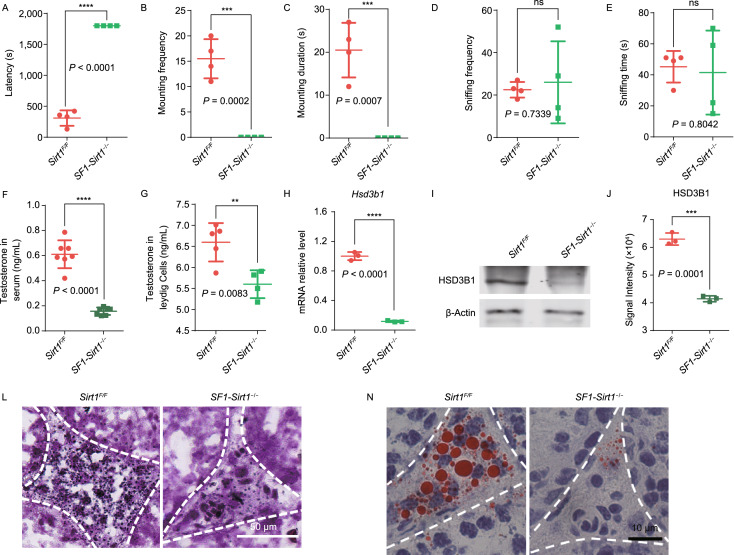

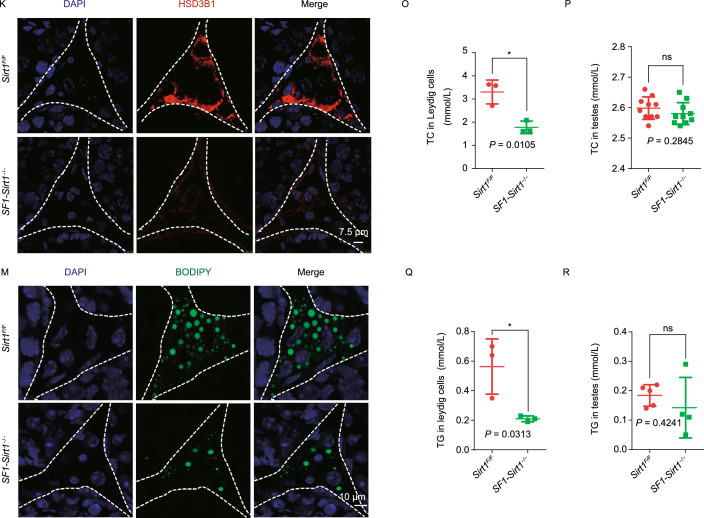
***Sirt1*** **disruption in Leydig cells affects sexual behavior and interrupts steroidogenic activity**. (A) The sexual behavior of *SF1-Sirt1*^−/−^ mice was severely compromised as these mice showed prolonged latencies in mounting target females compared to that of *Sirt1*^*F*/*F*^ mice. (B and C) Similarly, *SF1-Sirt1*^−/−^ mice mounted targeted females less frequently and also showed a significantly decreased mounting duration compared to *Sirt1*^*F*/*F*^ mice. (D and E) Sniffing frequency and sniffing time showed no obvious differences among *Sirt1*^*F*/*F*^ and *SF1-Sirt1*^−/−^ mice. (F) Testosterone levels in the sera of *SF1-Sirt1*^−/−^ mice showed a significant reduction compared to *Sirt1*^*F*/*F*^ mice. (G) Testosterone levels in the Leydig cells isolated from *SF1-Sirt1*^−/−^ mice were decreased significantly compared to the Leydig cells isolated from *Sirt1*^*F*/*F*^ mice. (H) The relative mRNA level of *Hsd3b1* was significantly decreased in the Leydig cells upon *Sirt1* disruption. (I) Immunoblotting analysis of HSD3B1 showed a significant reduction in *Sirt1*-deficient Leydig cells compared to those obtained from *Sirt1*^*F*/*F*^ mice. β-Actin was used as the loading control. (J) Quantification of the relative signal intensity of HSD3B1 shown in (I). Data being represented as mean ± SD and ****P < 0.0001. (K) Immunofluorescence staining showed a significant reduction in the signal of HSD3B1 in the Leydig cells from *SF1-Sirt1*^−/−^ mice compared to those of *Sirt1*^*F*/*F*^ mice. (L) The activity of the steroidogenic enzyme HSD3B1 was significantly reduced in the Leydig cells from *SF1-Sirt1*^−/−^ mice compared to those of *Sirt1*^*F*/*F*^ mice. (M) An extreme reduction in lipid content was found in the Leydig cells of *SF1-Sirt1*^−/−^ mice. BODIPY staining showed a sharp decrease in the number of LDs in the Leydig cells of *Sirt1*-deficient mice. (N) ORO staining performed on the testicular sections of *SF1-Sirt1*^−/−^ mice showed a significant reduction of LDs in the Leydig cells compared to *Sirt1*^*F*/*F*^ mice. (O) TC levels were significantly reduced in the Leydig cells of *SF1-Sirt1*^−/−^ mice compared to the Leydig cells of *Sirt1*^*F*/*F*^ mice. (P) No obvious differences were found in the TC levels in the whole testes extracts of *Sirt1*^*F*/*F*^ and *SF1-Sirt1*^−/−^ mice. (Q) TG levels were significantly reduced in the Leydig cells of *SF1-Sirt1*^−/−^ mice compared to the Leydig cells of *Sirt1*^*F*/*F*^ mice. (R) No obvious difference was found in the TG levels in the whole testes extracts of *Sirt1*^*F*/*F*^ and *SF1-Sirt1*^−/−^ mice

Different factors affect serum testosterone levels (Chen et al., 2009; Midzak et al., 2009). We firstly measured luteinizing hormone (LH) and follicle-stimulating hormone (FSH) concentrations in the sera of both *Sirt1*^*F*/*F*^ and *SF1-Sirt1*^−/−^ mice but found no significant differences in LH and FSH concentrations (Fig. S3A and S3B). Next, we focused on testosterone synthesis related factors. A large number of steroidogenic factors are involved in the transformation of cholesterol to testosterone, including LH receptor (LHCGR), lipoprotein receptors, cholesterol transporter STAR, and some steroidogenic enzymes CYP17A1, HSD3B1, CYP17A1 and HSD17B3 (Fig. S4A). Therefore, we measured the mRNA levels of these steroidogenic factors step by step from the initial raw material, cholesterol to testosterone, and found a significant decrease in these factors (Fig. S4). As HSD3B1 is an important enzyme involved in testosterone biosynthesis, we measured the mRNA and protein level of this enzyme and found a significant decrease in the levels of both mRNA and protein in the Leydig cells of *SF1-Sirt1*^−/−^ mice (Fig. 1H–J). Next, we assessed the levels of HSD3B1 in testicular sections by immunofluorescence and noted a significant decrease in the Leydig cells of *SF1-Sirt1*^−/−^ mice as compared to those of *Sirt1*^*F*/*F*^ mice (Fig. 1K). To further confirm these findings, we measured HSD3B1 activity and found a considerable decline in the Leydig cells isolated from *SF1-Sirt1*^−/−^ mice compared to control mice (Fig. 1L). Thus, these findings suggest perturbed steroidogenesis in *SF1-Sirt1*^−/−^ mice.

Cholesterol is utilized as major raw material to synthesize testosterone in the Leydig cells (Hu et al., 2010), therefore we carried out immunofluorescence analysis of BODIPY to detect the presence of cytoplasmic LDs, where cholesterol is stored as cholesterol esters (Ouimet et al., 2019). To our surprise, a significant decrease in LDs was observed in the testicular sections of *SF1-Sirt1*^−/−^ mice (Fig. 1M). Similar results were also obtained using Oil Red O (ORO) staining, where we found a clear decrease in LDs in *SF1-Sirt1*^−/−^ mice (Fig. 1N). We further assessed total cholesterol (TC) levels and found a significant decrease in Leydig cells isolated from *SF1-Sirt1*^−/−^ mice (Fig. 1O) but not in whole testis extracts or sera (Figs. 1P and S5A). Triglycerides (TG) are also stored in LDs, so we next measured TG concentrations in Leydig cells. Similar to TC, TG levels were also reduced significantly in the Leydig cells and sera (Figs. 1Q and S5B), but not whole testis extracts (Fig. 1R) isolated from *SF1-Sirt1*^−/−^ mice. Collectively, these results suggest that the testosterone deficiency in *SF1-Sirt1*^−/−^ mice might come from inadequate cholesterol transport into the Leydig cells.

Thus, decrease of LDs and cholesterol in the Leydig cells of *SF1-Sirt1*^−/−^ mice might be a result of decreased supplies of cholesterol. High-density lipoprotein (HDL) serves as the chief cholesterol source in testosterone biosynthesis in the Leydig cells. Therefore, we performed an *in vitro* cholesterol-uptake experiment employing fluorescence-labeled cholesterol-rich lipoprotein 1,1’-dioctadecyl-3,3,3’,3’-tetramethylindocarbocyanine perchlorate (DiI)-HDL in Leydig cells isolated from *Sirt1*^*F*/*F*^ and *SF1-Sirt1*^−/−^ mice to find out whether SIRT1 participates in cholesterol uptake from HDL. A significant decrease in cholesterol absorption was observed in the Leydig cells of *SF1-Sirt1*^−/−^ mice compared to *Sirt1*^*F*/*F*^ mice (Fig. 2A). Together, our findings suggest that SIRT1 actively participates in cholesterol uptake for steroidogenesis in Leydig cells.

**Figure 2:**
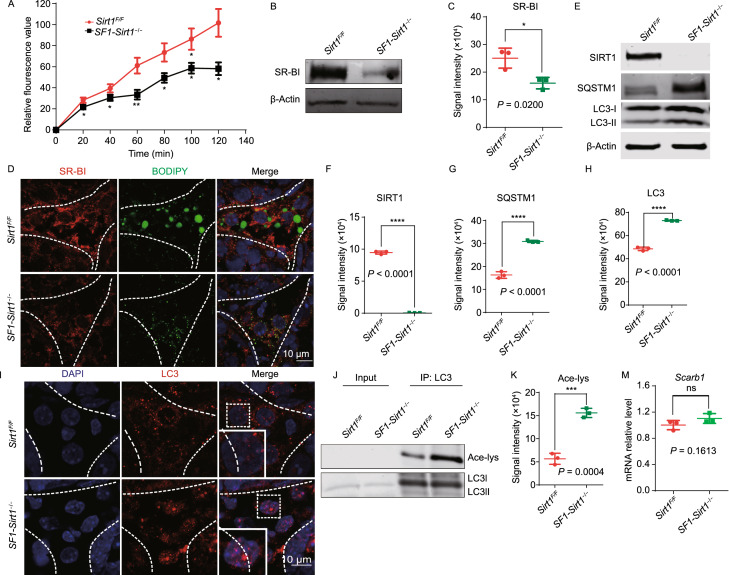

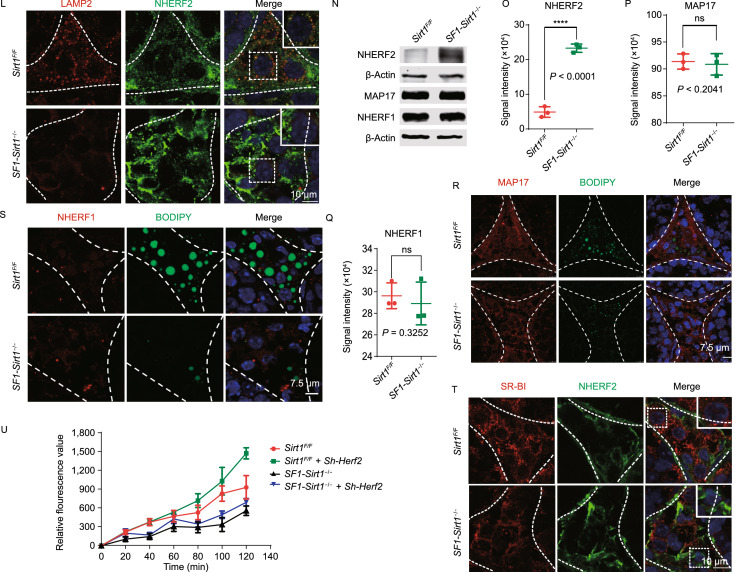
***Sirt1*** **disruption impairs autophagy and hinders cholesterol uptake in Leydig cells**. (A) Cholesterol (Dil-HDL) absorption was significantly reduced in the *Sirt1*-deficient Leydig cells compared to the Leydig cells of *Sirt1*^*F*/*F*^ mice. (B) Immunoblotting analysis of SR-BI showed a significant reduction of SR-BI in *Sirt1*-deficient Leydig cells compared to Leydig cells of *Sirt1*^*F*/*F*^ mice. β-Actin was used as the loading control. (C) Quantification of the signal intensity of SR-BI shown in (B). Data is represented as mean ± SD and *P < 0.0001. (D) Immunofluorescence staining of SR-BI showed a significant decrease in the Leydig cells of *SF1-Sirt1*^−/−^ mice compared to the Leydig cells of *Sirt1*^*F*/*F*^ mice. BODIPY staining showed a sharp decrease in the number of LDs in the Leydig cells of *Sirt1*-deficient mice compared to *Sirt1*^*F*/*F*^ mice. (E) SIRT1 levels were significantly reduced in autophagy-deficient Leydig cells compared with *Sirt1*^*F*/*F*^ Leydig cells. SQSTM1/p62 and LC3 showed considerable accumulation in the Leydig cells of *SF1-Sirt1*^−/−^ mice. (F) Quantification of the relative signal intensities of SIRT1 shown in (E). Data are represented as mean ± SD and ****P < 0.0001. (G) Quantification of the signal intensities of SQSTM1/p62 shown in (E). Data are represented as mean ± SD and ****P < 0.0001. (H) Quantification of the signal intensities of LC3 shown in (E). Data are represented as mean ± SD and ****P < 0.0001. (I) Immunofluorescence staining of LC3 showed a significant accumulation of LC3 puncta in the nuclei of Leydig cells of *SF1-Sirt1*^−/−^ mice. (J) Immunoprecipitation analysis to detect the acetylation level of endogenous LC3 in the Leydig cells of *Sirt1*^*F*/*F*^ and *SF1-Sirt1*^−/−^ mice. Western blot was used to analyze the immunocomplexes. (K) Quantification of the signal intensity of Ace-lys shown in (J). Data are represented as mean ± SD and ***P < 0.001. (L) Immunofluorescence staining of LAMP2 showed a significant decrease in the Leydig cells of *SF1-Sirt1*^−/−^ mice compared to the Leydig cells of *Sirt1*^*F*/*F*^ mice while immunofluorescence staining of NHERF2 showed a significant increase in the Leydig cells of *SF1-Sirt1*^−/−^ mice compared to the Leydig cells of *Sirt1*^*F*/*F*^ mice. (M) The relative mRNA levels of *Scarb1* were not significantly different in the Leydig cells of *Sirt1*^*F*/*F*^ and *SF1-Sirt1*^−/−^ mice upon *Sirt1* disruption. (N) Immunoblotting analysis of NHERF2 showed a significant accumulation in autophagy-deficient Leydig cells compared to the Leydig cells of *Sirt1*^*F*/*F*^ mice. However, no obvious difference was detected in the levels of NHERF1 and MAP17 in either the Leydig cells of *Sirt1*^*F*/*F*^ or *SF1-Sirt1*^−/−^ mice. β-Actin was used as the loading control. (O) Quantification of the signal intensity of NHERF2 shown in (N). Data being represented as mean ± SD and ****P < 0.0001. (P) Quantification of the signal intensity of MAP17 shown in (N). Data being represented as mean ± SD. (Q) Quantification of the signal intensity of NHERF1 shown in (N). Data being represented as mean ± SD. (R and S) Immunofluorescence analyses of MAP17 and NHERF1 showed no obvious differences between the Leydig cells of *Sirt1*^*F*/*F*^ and *SF1-Sirt1*^−/−^ mice, whereas BODIPY staining showed a sharp decrease in the number of LDs in the Leydig cells of *Sirt1*-deficient mice compared to *Sirt1*^*F*/*F*^ mice. (T) Immunofluorescence staining of SR-BI showed a significant decrease whereas NHERF2 showed a significant increase in the Leydig cells of *SF1-Sirt1*^−/−^ mice compared to the Leydig cells of *Sirt1*^*F*/*F*^ mice. (U) Cholesterol (Dil-HDL) absorption was significantly reduced in the *Sirt1*-deficient Leydig cells compared to the Leydig cells of *Sirt1*^*F*/*F*^ mice. However, *Nherf2*-knockdown partially rescued the Cholesterol (Dil-HDL) absorption defect in the Leydig cells isolated from *SF1-Sirt1*^−/−^ mice

SR-BI has been described as a major lipoprotein receptor specifically involved in the uptake of HDL, LDL, or VLDL. Therefore, to explore whether SR-BI participates in cholesterol absorption, we measured SR-BI levels via immunoblotting and found a significant decrease in SR-BI levels in the Leydig cells isolated from *SF1-Sirt1*^−/−^ mice compared to control mice (Fig. 2B and 2C). To further confirm these findings, we carried out immunofluorescence analysis of SR-BI in testicular sections. A significant reduction in SR-BI signal was found in *SF1-Sirt1*^−/−^ testes compared to control testes (Fig. 2D). Together, reduced cholesterol in the Leydig cells of *SF1-Sirt1*^−/−^ mice might represent perturbed cholesterol uptake as a result of SR-BI down-regulation.

SIRT1 has been reported to be an important regulator of autophagy by directly deacetylating LC3 and ATG7, thus affects their translocation from the nucleus to the cytoplasm (Chang et al., 2015). Therefore, we investigated the effects of *Sirt1*-knock out on autophagy flux and found that SQSTM1/p62 and LC3I (cytosolic form), but not LC3II (membrane-bounded form), accumulated in Leydig cells (Fig. 2E–H), suggesting that autophagic flux is indeed disrupted in the Leydig cells of *SF1-Sirt1*^−/−^ mice. To test the nucleocytoplasmic redistribution of LC3, we carried out immunofluorescence analysis of testicular sections from *Sirt1*^*F*/*F*^ and *SF1-Sirt1*^−/−^ mice and found a clear accumulation of LC3 in the nuclei of *Sirt1*-deficient Leydig cells (Fig. 2I). To further verify our immunofluorescence results, we examined the LC3 acetylation level in the Leydig cells isolated from *Sirt1*^*F*/*F*^ and *SF1-Sirt1*^−/−^ mice and found a significant increase in LC3 acetylation in the Leydig cells of *Sirt1*-deficient mice (Fig. 2J and 2K). Thus, SIRT1 might regulate the autophagic process by modulating LC3 acetylation. Decrease in LAMP2 (marker of lysosomes or autolysosomes presence) levels has also been reported in the absence of SIRT1 (Shi et al., 2018). Therefore, we assessed LAMP2 levels by immunofluorescence and found a clear decrease in LAMP2 levels in *Sirt1*-deficient Leydig cells (Fig. 2L). Collectively, these results suggest a disruptive autophagic process in the steroidogenic cells of *SF1-Sirt1*^−/−^ mice, and the down-regulation of SR-BI might also come from the disruption of autophagic flux. To find out how SR-BI is regulated in steroidogenic cells via autophagy, relative mRNA levels of *Scarb1* was measured in both *Sirt1*^*F*/*F*^ and *SF1-Sirt1*^−/−^ Leydig cells. Surprisingly, *Scarb1* mRNA was not decreased but was instead slightly increased in *SF1-Sirt1*^−/−^ Leydig cells (Fig. 2M), suggesting SIRT1 might regulate SR-BI via degradation of its negative regulators. MAP17, NHERF1, and NHERF2 are known negative regulators of SR-BI. Therefore, we first measured the levels of these proteins by immunoblotting and found a significant accumulation of NHERF2 in *SF1-Sirt1*^−/−^ Leydig cells (Fig. 2N and 2O), while the levels of MAP17 and NHERF1 was comparable between *Sirt1*^*F*/*F*^ and *SF1-Sirt1*^−/−^ Leydig cells (Fig. 2N, 2P, and 2Q). To verify these findings, we performed immunofluorescence analysis and found no difference between the signals of MAP17 and NHERF1 in *Sirt1*^*F*/*F*^ and *SF1-Sirt1*^−/−^ mouse testes (Fig. 2R and 2S) but detected a significant increase in NHERF2 in *SF1-Sirt1*^−/−^ mouse testes compared with control mouse testes (Fig. 2L). To further investigate the relationship of these proteins, we checked SR-BI and NHERF2 levels in Leydig cells via immunofluorescence. NHERF2 and SR-BI signals were negatively correlated in *Sirt1*^*F*/*F*^ Leydig cells, whereas NHERF2 accumulation led to a considerable reduction in SR-BI level in *SF1-Sirt1*^−/−^ Leydig cells (Figs. 2T and S6). Because NHERF2 has previously been reported to be selectively degraded by the autophagy-lysosome pathway (Gao et al., 2018), our new results further suggest that the SIRT1-dependent autophagy-lysosome pathway should be involved in NHERF2 degradation in Leydig cells.

Next, to check whether NHERF2 accumulation is responsible for cholesterol uptake defect observed in *SF1-Sirt1*^−/−^ Leydig cells, we tested whether *Nherf2*-knockdown could rescue cholesterol uptake defect in *SF1-Sirt1*^−/−^ Leydig cells. Therefore, we measured cholesterol uptake in *Nherf2*-knockdown *SF1-Sirt1*^−/−^ and *Sirt1*^*F*/*F*^ Leydig cells, and found a clear increase in both rates and amounts of cholesterol uptake, as shown by DiI-HDL absorption, compared to *Nherf2*-expressing cells (Fig. 2U). Collectively, NHERF2 seems to act as a connecter between SIRT1 and cholesterol absorption in testicular steroidogenic cells. And, SIRT1 impairment in Leydig cells leads to abnormal accumulation of NHERF2 that downregulates SR-BI, leading to impaired cholesterol uptake and insufficient testosterone biosynthesis. It is noteworthy to mention that decrease in testosterone level was consistent with a significant decrease in steroidogenesis activity and steroidogenesis at the transcriptional and translational level in *SF1-Sirt1*^−/−^ Leydig cells (Figs. 1H–J and S4), and several steroidogenesis factors were also downregulated in *Sirt1* conventional knockout mice (Kolthur-Seetharam et al., 2009). As the expression of some steroidogenic enzymes could also be partially rescued in *Nherf2*-knockdown *SF1-Sirt1*^−/−^ Leydig cells (Fig. S7), the dysfunction of the testosterone synthesis factors might occur in response to the insufficient cholesterol supply. Besides, SIRT1 has been reported to participate in several cellular processes such as gene silencing, glucose and lipid metabolism (Cohen et al., 2004; Chang et al., 2015), SIRT1 may have extensive and multiple functions in the manifold regulation of Leydig cells.

Taken together, SIRT1 deacetylates LC3 in the nucleus, and eventually, LC3 moved from the nucleus to the cytoplasm, where LC3 participates in autophagosome formation by interacting with the other components of the autophagic machinery. NHERF2 is taken up and ultimately degraded via autophagosomes, thus promoting SR-BI expression and accelerating the uptake of cholesterol to fuel the process of steroidogenesis. While in the absence of SIRT1, LC3 fails to deacetylate and translocate to the cytoplasm. Due to autophagic flux disruption, NHERF2 remains intact, thus inhibiting SR-BI expression and cholesterol cannot be efficiently up taken by the Leydig cells, therefore testosterone cannot be synthesized and finally results in LOH (Fig. S8).

## Electronic supplementary material

Below is the link to the electronic supplementary material.

Supplementary material 1 (PDF 1758 kb)
