# Supplementary material for: Sirt1 regulates testosterone biosynthesis in Leydig cells via modulating autophagy
Source: Protein Cell. 2020 Oct 13;12(1):67–75. doi: 10.1007/s13238-020-00771-1 (PMC7815870; doi:10.1007/s13238-020-00771-1)
Supplement: Supplementary file 1 — Supplementary material 1 (PDF 1758 kb) [file 13238_2020_771_MOESM1_ESM.pdf]

Supplemental materials

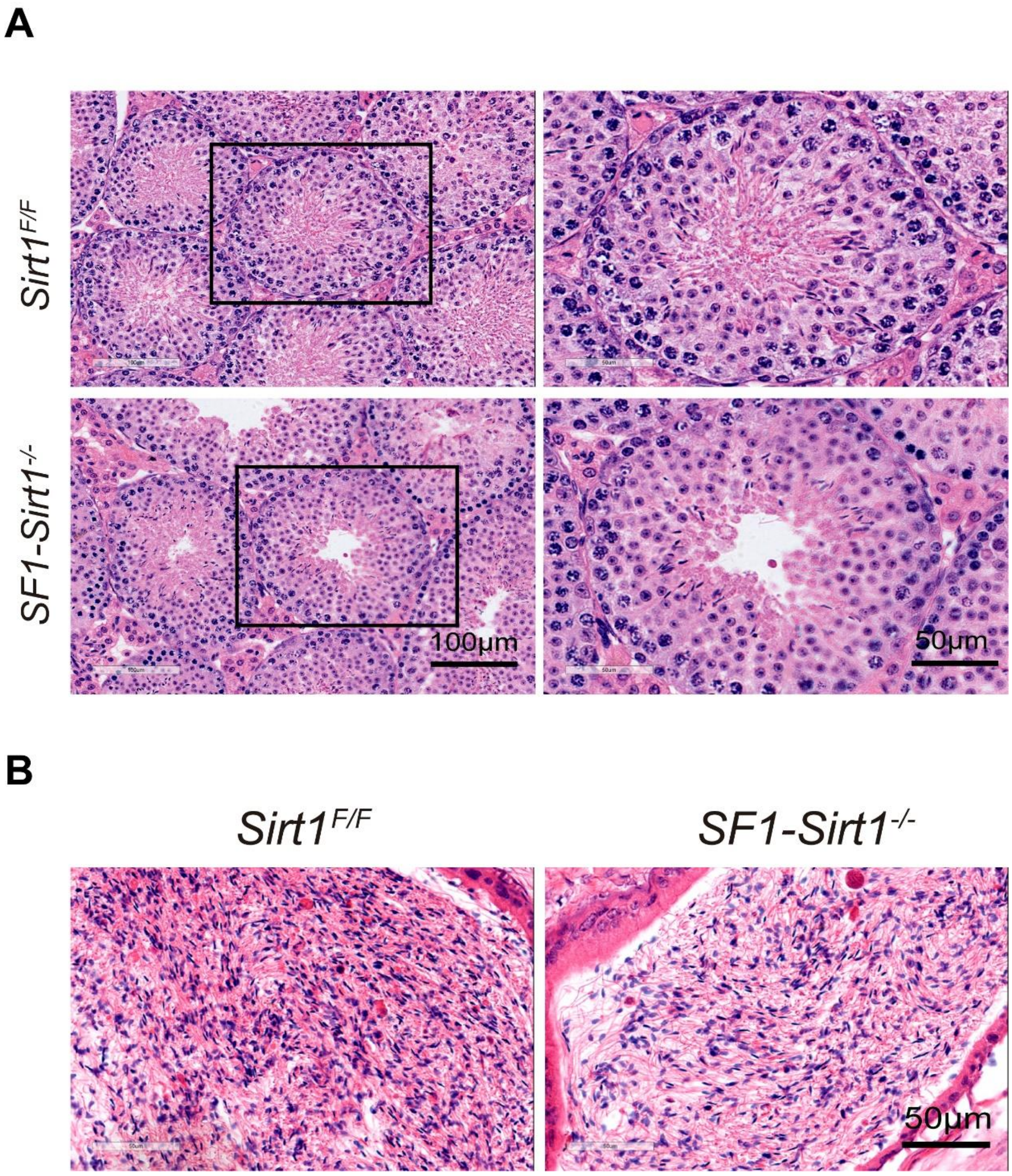

Supplemental Figure 1: Steroidogenic cell-specific *Sirt1*-knockout did not affect spermatogenesis. (A) H & E stained sections of testes of 8 weeks old *Sirt1*$^{F/F}$ and *SF1-Sirt1*$^{-/-}$

mice. **(B)** H & E stained sections of cauda epididymis of 8 weeks old *Sirt1*$^{F/F}$ and *SF1-Sirt1*$^{-/-}$ mice.

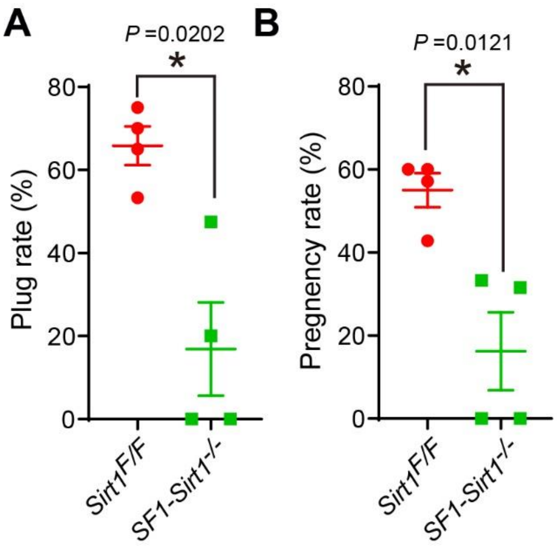

Supplemental Figure 2: Steroidogenic cell-specific *Sirt1*-knockout causes male subfertility. (A) The mating efficiency of *SF1-Sirt1*$^{-/-}$ male mice was reduced compared with *Sirt1*$^{F/F}$ male mice. **(B)** The fertility of *SF1-Sirt1*$^{-/-}$ male mice was reduced compared with *Sirt1*$^{F/F}$ male mice.

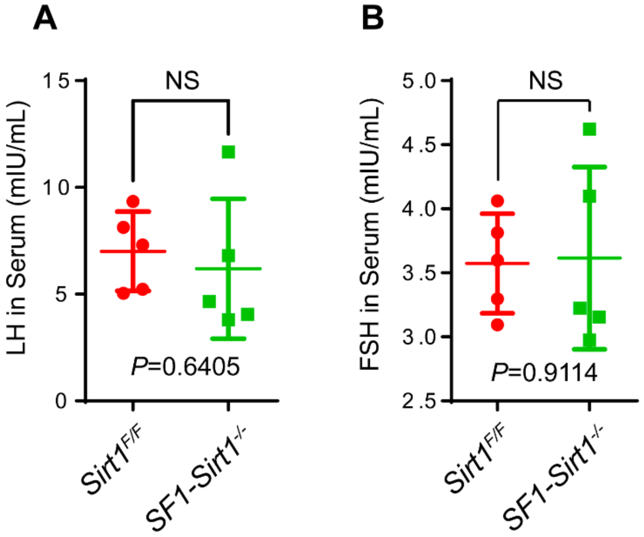

Supplemental Figure 3: *Sirt1*-knockout in Leydig cells did not affect LH and FSH levels. (A) LH levels in the sera of both *Sirt1*$^{F/F}$ and *SF1-Sirt1*$^{-/-}$ mice showed no significant difference. **(B)** FSH levels in the sera of both *Sirt1*$^{F/F}$ and *SF1-Sirt1*$^{-/-}$ mice showed no significant difference.

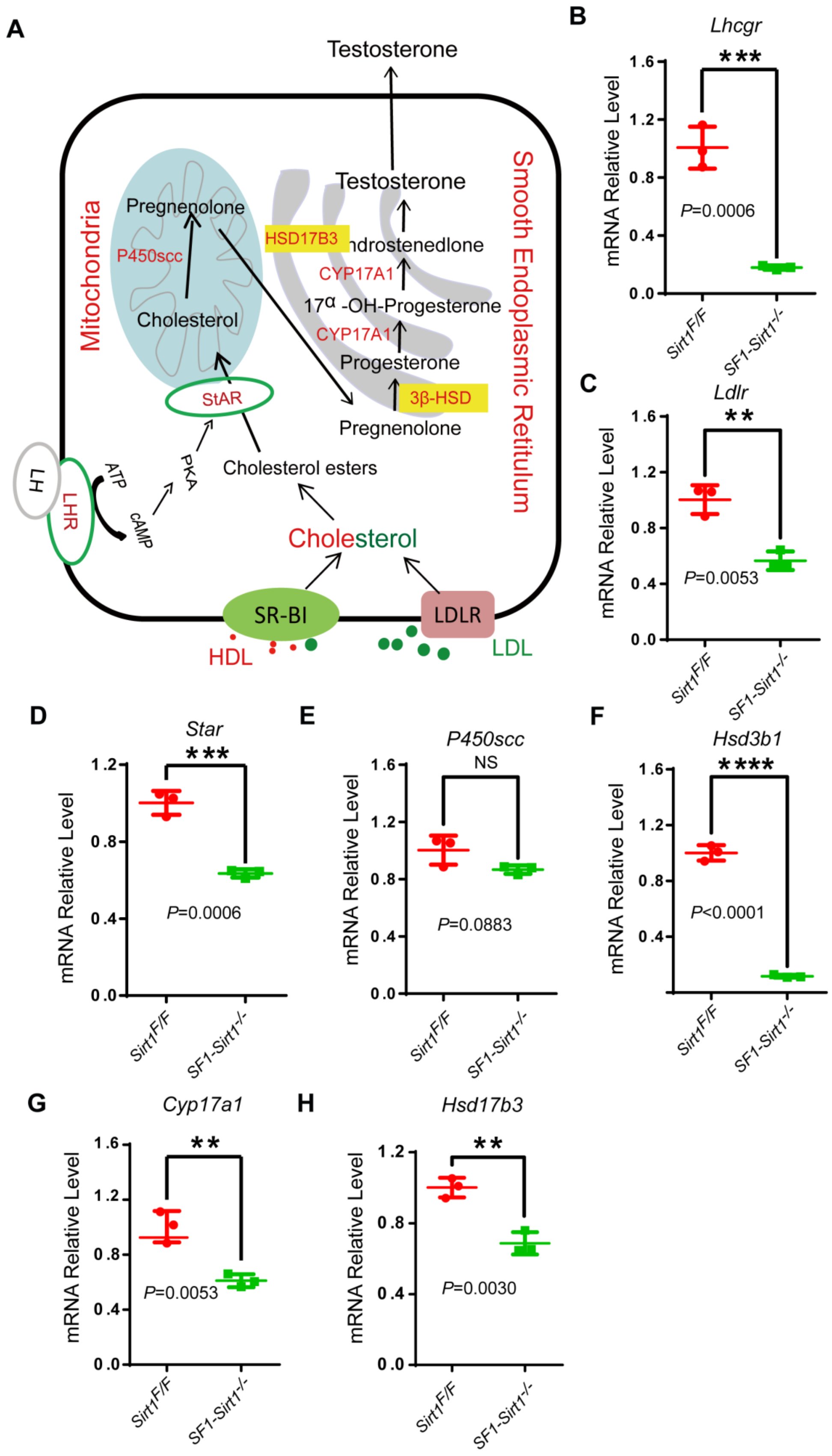

A
Testosterone
Mitochondria
Smooth Endoplasmic Retitulum
Pregnenolone
P450scc
Cholesterol
HSD17B3
Androstenedlone
CYP17A1
17α -OH-Progesterone
CYP17A1
Progesterone
3β-HSD
Pregnenolone
StAR
PKA
ATP
cAMP
LH
LHR
Cholesterol esters
Cholesterol
SR-BI
LDLR
HDL
LDL
B
Lhcgr
***
P=0.0006
C
Ldlr
**
P=0.0053
D
Star
***
P=0.0006
E
P450scc
NS
P=0.0883
F
Hsd3b1
****
P<0.0001
G
Cyp17a1
**
P=0.0053
H
Hsd17b3
**
P=0.0030
mRNA Relative Level
Sirt1F/F
SF1-Sirt1-/-

Supplemental Figure 4: *Sirt1*-knockout affects the expression of several steroidogenic factors in the Leydig cells. (A) Schematic of testosterone biosynthesis pathways in Leydig cells. **(B-H)** Real-time quantitative PCR (qPCR) analysis revealed the relative mRNA level of several important steroidogenic factors involved in the process of steroidogenesis were downregulated in the Leydig cells upon steroidogenic cell-specific *Sirt1*-knockout.

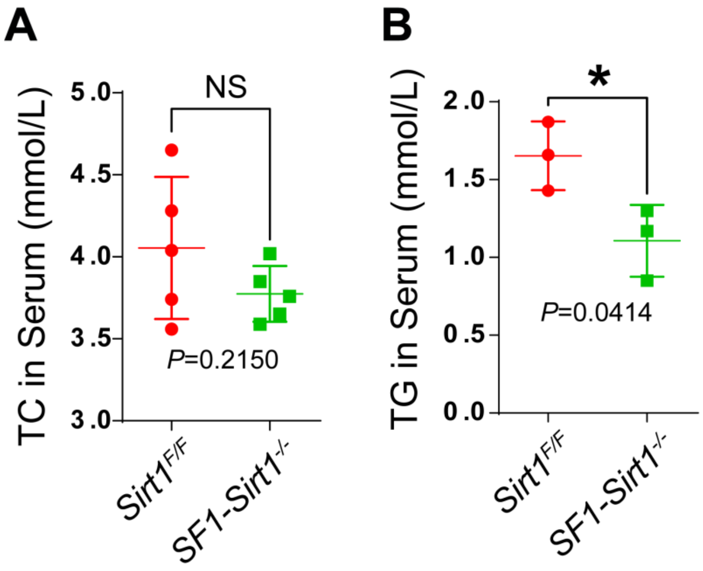

Supplemental Figure 5: TC and TG in the sera of *Sirt1*$^{F/F}$ and *SF1-Sirt1*$^{-/-}$ mice. (A) No obvious differences were found in the levels of TC in the sera of *Sirt1*$^{F/F}$ and *SF1-Sirt1*$^{-/-}$ mice. **(B)** TG levels were significantly reduced in the sera of *SF1-Sirt1*$^{-/-}$ mice compared to the sera of *Sirt1*$^{F/F}$ mice.

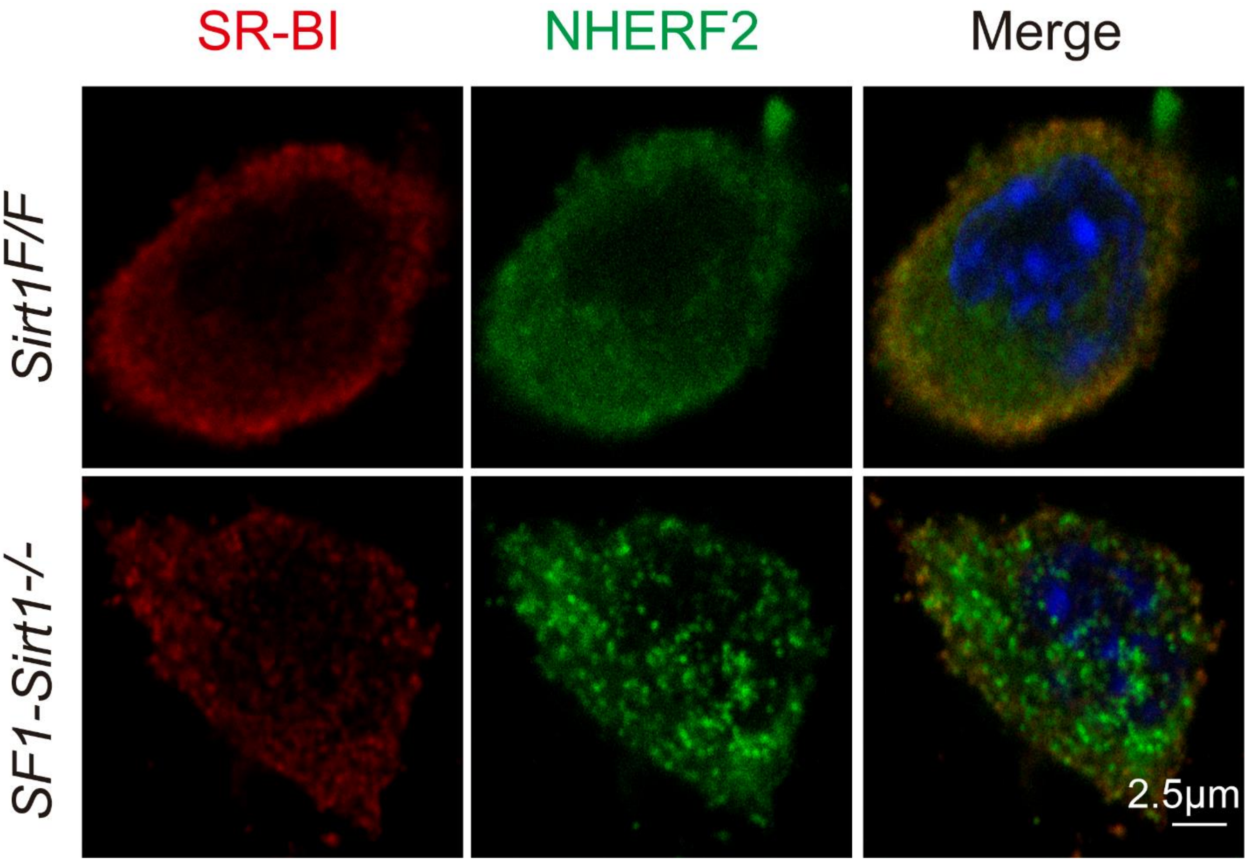

Supplemental Figure 6: Immunofluorescence staining of SR-BI and NHERF2 in the Leydig cells isolated from *Sirt1*$^{F/F}$ and *SF1-Sirt1*$^{-/-}$ mice. Immunofluorescence staining of SR-BI showed a significant decrease whereas NHERF2 showed a significant increase in the Leydig cells isolated from *SF1-Sirt1*$^{-/-}$ mice compared to the Leydig cells isolated from *Sirt1*$^{F/F}$ mice.

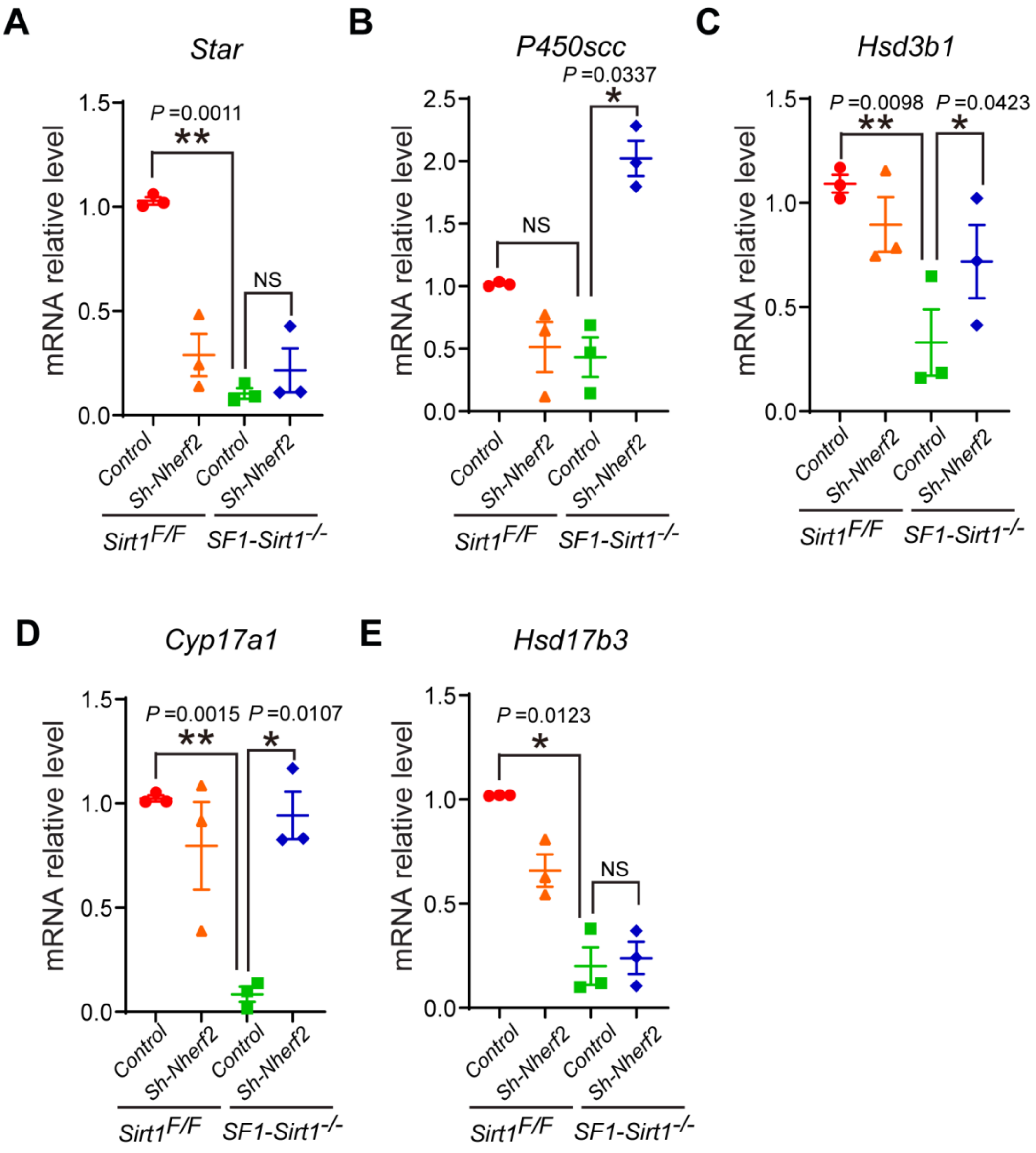

Supplemental Figure 7: The expression of some steroidogenic factors could be partially rescued in *Nherf2*-knockdown *SF1-Sirt1*$^{-/-}$ Leydig cells. Real-time quantitative PCR (qPCR) analysis was performed to detect the relative mRNA level of several important steroidogenic factors involved in the process of steroidogenesis in *SF1-Sirt1*$^{-/-}$ and *Sirt1*$^{F/F}$ Leydig cells treated with *Sh-Nherf2* or not.

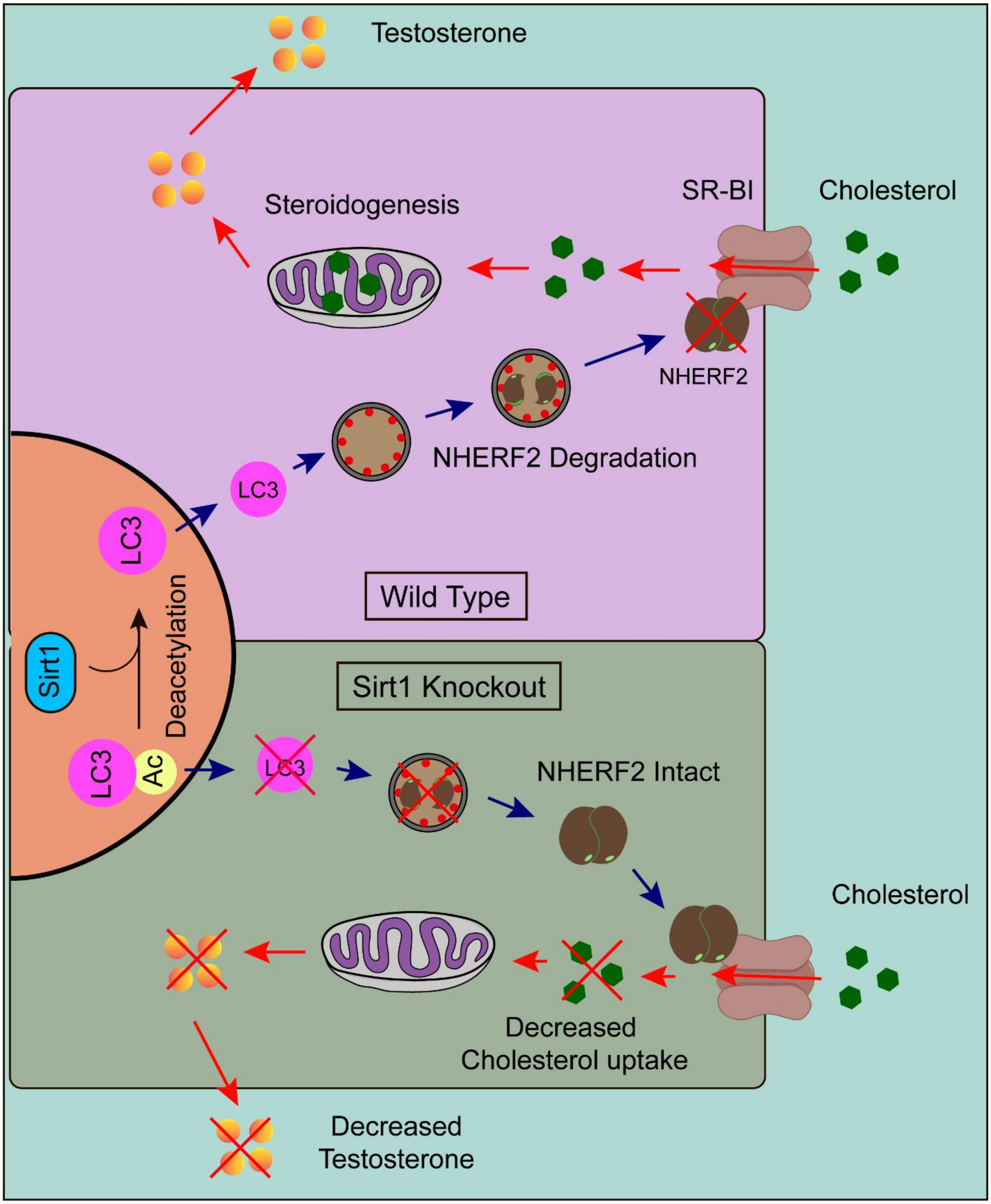

Supplemental Figure 8: Schematic of how SIRT1 regulates testosterone biosynthesis in Leydig cells via modulating autophagy. SIRT1 performs deacetylation of LC3 in the nucleus

and eventually, LC3 moved from the nucleus to the cytoplasm, where it participates in autophagosome formation by interacting with the other components of the autophagic machinery. NHERF2 is a negative regulator of SR-BI which regulates cholesterol uptake in the Leydig cells. This negative regulator could be taken up and ultimately degraded via selective autophagy, hence promoting the uptake of cholesterol to fuel the process of steroidogenesis. However, in the absence of SIRT1, LC3 fails to deacetylate and move to the cytoplasm and participates in autophagosome biogenesis. Therefore, NHERF2 remains intact and hinders the cholesterol uptake by SR-BI, resulting in insufficient cholesterol supplies and testosterone biosynthesis.

Supplementary Table 1: List of primers used for real-time quantitative PCR (qPCR) analyses

Gene	Forward primer（5’3’）	Reverse primer (5’-3’)
Hsd17b3	ATGGCATCGGGAAAGCCTAT	CTCTTCTGCAATGGTCTGTAG C
Cyp17a1	GATCTAAGAAGCGCTCAGGCA	GGGCACTGCATCACGATAAA
Hsd3b1	CTCAGTTCTTAGGCTTCAGCAA TTAC	CCAAAGGCAAGATATGATTTA GGA
P450scc	CCAGTGTCCCCATGCTCAAC	TGCATGGTCCTTCCAGGTCT
Star	CCGGAGCAGAGTGGTGTCA	CAGTGGATGAAGCACCATGC
Lhr	AATGAGTCCATCACGCTGAAAC	CCTGCAATTTGGTGGAAGAGA
Gapdh	AGAACATCATCCCTGCATCCA	CCGTTCAGCTCTGGGATGAC
Scarb1	GCCCATCATCATCTGCCAACT	TCCTGGGAGCCCTTTTTACT
Ldlr	CCCTCAAGACAGATGGTC	CAGCCCAGCTTTGCTCTTAT

Materials and Methods

Mice

Sirt1$^{F/F}$ and *SF1-Sirt1*$^{-/-}$ mice were obtained and further bred from *Sirt1*$^{F/F}$ and SF1-Cre mouse strains (Jackson Laboratory). All the mice employed in the current study were kept and reared under controlled environmental conditions with 12 hours of illumination and provision of free access to food and water. All the experimental procedures involving animals were reviewed and approved by the Animal Research Panel of the Committee on Research Practice of the University of Chinese Academy of Sciences, Beijing, P.R. China.

Antibodies

The following antibodies were utilized: rabbit antibodies against LC3 (L7543-100UL, Sigma),

SQSTM1/p62 (5114S, Cell Signaling), acetylated-lysine (9441, Cell Signaling), SIRT1 (07-1310 Merck), LC3 (ab58610, Abcam), SR-BI (NB400-104, Novus), NHERF1 (SC-134485, Santa Cruz Biotechnology), rat antibodies against LAMP2 (ab13524, Abcam), goat antibodies against MAP17 ((SC-27375, Santa Cruz Biotechnology), mouse antibodies against NHERF2 (SC-365388, Santa Cruz Biotechnology), 3β-HSD (SC-515120, Santa Cruz Biotechnology), FITC and TRITC-conjugated secondary antibodies against rabbit and mouse (Zhong Shan Jin Qiao, Beijing, China). Alexa Fluor 680-conjugated goat anti-mouse secondary antibodies (western blot analysis) were purchased from Invitrogen.

Other chemicals and reagents

Dulbecco's Modified Eagle Medium–Ham’s nutrient mixture F-12 (F12/DMEM; SH30023.01B) was purchased from HyClone. HBSS (C141755001BT) lacking Ca^{2+}, $Mg^{2+,}$ and penicillin-streptomycin (15140–122) was bought from Gibco. Collagenase type IV

(C5138) was purchased from Sigma-Aldrich. Percoll (sc-296039) was supplied by Santa Cruz Biotechnology, Inc. DiI-HDL (H8910) was obtained from Solarbio. Radioimmunoprecipitation assay (RIPA) buffer (P0013C) was supplied by Beyotime. TG (F001-1) and TC kits (F002-1) were bought from Nanjing Jiancheng Bioengineering Institute, and BOD IPY 493/503 (D3922) was bought from Invitrogen.

Mating and fertility assay

The sexual behaviors of *Sirt1*$^{F/F}$ and *SF1-Sirt1*$^{-/-}$ mice were analyzed as described previously (Gao et al., 2018). Briefly, a 6-wk-old sexually naive estrous CD1 female mouse was caged with each of the test male mice. Each assay lasted for 30 minutes after the introduction of the targeted mice. An infrared camera was used to record all the activities. Latency, sniffing and mounting duration and frequency of the male mouse were examined. Assessment of the fertility of *Sirt1*$^{F/F}$ and *SF1-Sirt1*$^{-/-}$ mice were analyzed as described previously. Briefly, males of different genotypes (8-9 weeks) were used for the breeding assay. Each male mouse was caged with two wild-type CD1 females (7-8 weeks), and their vaginal plugs were checked every morning. The rate of pregnancy was counted. Each male underwent four cycles of the above breeding assay with different females.

Immunofluorescence staining

Immediately after the dissection, testes were harvested and quickly embedded in an optimum cutting temperature compound (OCT) (4583; Tissue-Tek) and 5 μm thick sections were cut using a cryo-microtome (CM1950; Leica Biosystems). Sections were mounted on slides and fixed using 4% PFA (P1110; Solarbio). These fixed sections were washed with PBS three times and blocked with 5% *bovine serum albumin* having 0.1% Triton X-100. After blocking, the testicular sections were immersed in primary antibody for 12 hours at 4 ℃ and then incubated with a

secondary antibody at 37 ℃ for 1-2 hours. DAPI was used to stain the nuclei. All the images were captured using a TCS SP8 microscope (Leica Microsystems). Image adjustments were made to make images suitable for publication using Adobe Photoshop CC (2018).

Leydig cells were collected and cultured onto a thin cover glass for 12 hours to analyze cellular immunofluorescence. PBS (pH 7.4) was used to rinse the cells three times, and the cells were fixed and stained as described above.

The frozen sections were fixed using 4% PFA, followed by rinsing three times with PBS (pH 7.4), subsequently incubated with 1 μg/ml BODIPY–PBS solution for 10 min at RT, and processed for image acquisition as detailed above.

ORO staining

ORO staining was performed as reported previously (Gao et al., 2018). Briefly, 5 μm thick frozen testes sections were cut and fixed using 4% PFA for 15 min followed by rinsing three times with PBS (pH 7.4). After rinsing, sections were incubated in 60% (vol/vol) isopropyl alcohol for three minutes and air-dried. Subsequently, the air-dried sections were stained for 15 min using ORO solution as reported elsewhere (Bilińska, 1994). Next, to remove the background staining, the slides were rinsed using 70% ethanol for five seconds. Subsequently, the slides were rinsed with tap water and counterstained using Harris hematoxylin. Glycerol/ PBS (9:1) was used to mount tissues and then processed further.

Isolation and primary culture of Leydig cells

Leydig cells were separated as reported previously (Gao et al., 2018). Briefly, two-months-old mice were sacrificed, and gonads were collected and rinsed using prechilled PBS. Next, tunica albuginea was removed and de-capsulated testes were incubated in a shaking water bath at 37 ℃

for 15 minutes in a solution of 1mg/ml collagenase IV in DMEM/F12 medium with vibrations at 120rpm. Pre-chilled culture medium was added to stop the action of the collagenase. Seminiferous tubules settled to the bottom of the tube due to gravity sedimentation and interstitial cells remained suspended in the supernatant. The interstitial cells in the supernatant were isolated by centrifugation at 500g for 6 minutes and resuspended in 2 ml DMEM/F12 medium. These resuspended cells were loaded on the top of discontinuous Percoll gradients having 30%, 40%, 50%, and 60% of Percoll dissolved in HBSS followed by centrifugation at 600g at 25 ℃ for 28 minutes to collect the Leydig cells. Most of the Leydig cells were found to be distributed between 50% to 60% fractions. Leydig cells were removed carefully with a micropipette and rinsed three times with PBS following centrifugation at 600g for 6 minutes each. Histochemical staining with 3β-HSD confirmed >90% purity of the Leydig cells. Finally, the purified Leydig cells were used to perform western blotting and cultured in DMEM/F12 medium using conditions described previously (Gao et al., 2018).

Hormone Assessment

The level of LH and FSH in the sera were examined with a ready-to-use RIA kit (Beijing Sino-UK Institute of Biological Technology). Similarly, the level of testosterone in the sera and isolated Leydig cells was assessed using a ready-to-use RIA kit (Beijing Sino-UK Institute of Biological Technology).

TG and TC analyses

For TG and TC assessment in testes and Leydig cells, mice were sacrificed, and testes were harvested and decapsulated. Next, testes or isolated Leydig cells were homogenized as described elsewhere (Liu et al., 2017). To collect the supernatant, the homogenates were allowed to stand at room temperature for 15 minutes and centrifuged at 12,000 rpm for 10 minutes. Next, to

extract the total lipids from the supernatant, a 2:1 chloroform/methanol (vol/vol) mixture was added and incubated for 4 h at 37 ℃, followed by centrifugation at 3,000 rpm for 10 minutes. Subsequently, the organic layers in the bottom of the tubes were collected and evaporated with nitrogen gas overnight to reveal a pellet. The resulting pellet was resolubilized with double-distilled H_2O to measure TG and TC using ready-to-use commercial kits (TG kit-F001-1 and TC kit-F002-1; Nanjing Jiancheng Bioengineering Institute).

Cholesterol uptake experiment and time-lapse imaging

Leydig cells were isolated and plated in gelatin-coated chambers and cultured for 20 hours as described previously. To knockdown *Nherf2*, we cloned *Nherf2*-shRNA oligonucleotide (5′-GGG TTC AAC CTG CAT AGT GAC-3′) into the HpaI and XhoI sites of the pSicoR-GFP lentiviral vector (11579; Addgene). This lentiviral plasmid was co-transfected with the packaging plasmids pMD2.G (12259) and psPAX2 (12260; Addgene) into 293T cells and the desired virus was harvested after 48 hours of transfection. Leydig cells were infected with the virus using 1 mg/ml polybrene and incubated for 12 hours. The culture medium was replaced with 20 μg/ml DiI-HDL just before image acquisition, and Leydig cells were positioned on an UltraVIEW-VoX confocal laser microscope (PerkinElmer). Images were captured every 10 minutes for about 2 hours and fluorescence intensities of Leydig cells were compared and analyzed every 20 minutes using Volocity software (6.0; PerkinElmer).

Histochemical Staining for HSD3B1 Activity

Histochemical staining to assess HSD3B1 activity was performed according to a method reported previously (Klinefelter et al., 1987). Briefly, frozen testicular sections were washed with PBS three times for 5 minutes each time. Solution A (0.6 mg of DHEA dissolved in 0.3 ml DMSO), solution B (1 mg of NBT dissolved in 0.3 ml DMSO), and solution C (10 mg β-NAD

and 9.5 ml DPBS) were mixed. The sections were incubated with a mixture of A, B, and C solutions at 37 ℃ for 2 hours. After incubation, the sections were washed three times with PBS and stained with Hematoxylin for 3 minutes to visualize the nucleus. Glycerol was used for mounting, and slides were processed to capture the images.

Immunoblotting

Leydig cells were digested adopting a method described previously (Liu et al., 2016). The resulting homogenates were centrifuged for 20 minutes at 12000 rpm, supernatants were collected and analyzed using a protein assay from Bio-Rad Laboratories to determine protein concentrations. Protein lysates thus obtained were separated via SDS-PAGE and finally electro-transferred onto a nitrocellulose membrane. The membranes were incubated with 5% (w/v) BSA or nonfat milk and stained with the respective primary and secondary antibodies. The membranes were washed with PBS and finally scanned using an ODYSSEY Sa infrared imaging system (LI-COR Biosciences).

Real-time quantitative PCR (qPCR) analyses

The RNA samples were isolated from both *Sirt1*$^{F/F}$ and *SF1-Sirt1*$^{-/-}$ mice with an RNeasy Mini kit (74104; QIA GEN) according to the instructions manual. The RNA thus obtained was subjected to the action of DNase (RNase-free DNase set; QIA GEN) and was quantified via the photometric method (BioPhotometer). cDNA was prepared following the instruction manual of the Prime Script reverse transcription reagent kit (RR037A; Takara Bio Inc.). Real-time PCR was performed using a Light Cycler 480II system (Roche), and Light Cycle480 SW software (1.5.1; Roche) was used to analyze the results. Briefly, iQ SYBR green supermix (170-8882, Bio-Rad Laboratories) was used to prepare the samples, and PCR cycles were run using the following conditions: 95 ℃ for 10s, 60–62 ℃ for 45s, 95 ℃ for 60s, and 55 ℃ for 60s, followed

by a melting curve from 55–95 ℃ in steps of 0.5 ℃ and then subsequently held at 4 ℃ (iCycler iQ; Bio-Rad Laboratories) as described previously (Gao et al., 2018). To balance any potential irregularities in RNA concentration, all values were normalized to GAPDH. A negative control (RT−) was also used in each qPCR assay to control the efficacy of the process. The 2ΔΔCt method was adopted to measure the fold changes in gene expression. The complete list of sequences of primer pairs used is provided in Table S1.

Immunoprecipitation

Leydig cells were isolated and digested using a Dounce homogenizer (1234F35, Thomas Scientific) in a lysis buffer described previously (Liu et al., 2017). The resulting lysates were incubated with respective antibodies at 4 ℃ overnight, followed by incubation with protein A-Sepharose (GE Healthcare, 17-1279-03) for 3 h at 4 ℃. Next, the obtained immune complexes were subjected to washing five times using a lysis buffer and finally analyzed by immunoblotting.

Statistical analysis

All the experiments were repeated at least three times and the results are presented as the mean ± SD or SEM. The statistical significance of the differences among different groups was measured using the Student's t-test. The data were considered significant when the p-value was <0.05 (*) or <0.01 (**), <0.001 (***), <0.0001 (****).
